# Prevalence, Patterns, and Clinical Severity of Long COVID among Chinese Medicine Telemedicine Service Users: Preliminary Results from a Cross-Sectional Study

**DOI:** 10.3390/ijerph20031827

**Published:** 2023-01-19

**Authors:** Fai Fai Ho, Shanshan Xu, Talos Ming Hong Kwong, Angus Siu-cheong Li, Eun Hae Ha, Heyu Hua, Ching Liong, Kwan Chi Leung, Ting Hung Leung, Zhixiu Lin, Samuel Yeung-Shan Wong, Faming Pan, Vincent Chi Ho Chung

**Affiliations:** 1 School of Chinese Medicine, Faculty of Medicine, The Chinese University of Hong Kong, Shatin 999077, Hong Kong; 2 The Jockey Club School of Public Health and Primary Care, The Chinese University of Hong Kong, Shatin 999077, Hong Kong; 3 Department of Epidemiology & Biostatistics, School of Public Health, Anhui Medical University, Hefei 230032, China; 4 The Chinese University of Hong Kong Chinese Medicine Specialty Clinic cum Clinical Teaching and Research Centre, School of Chinese Medicine, Faculty of Medicine, The Chinese University of Hong Kong, Shatin 999077, Hong Kong

**Keywords:** COVID-19, SARS-CoV-2, activities of daily living, symptoms, phenotype, prevalence, cross-sectional study, C19-YRS, Post COVID-19 Syndrome

## Abstract

Introduction: The emergence and persistence of symptoms after acute COVID-19 is expected to become a major burden on healthcare systems. We assessed the features of the post-COVID-19 Syndrome (Long COVID) burden in a cohort of COVID-19 patients during the fifth major wave in Hong Kong. Methods: A cross-sectional study of 135 patients with confirmed COVID-19 from Feb to Apr 2022 who utilized traditional Chinese medicine telemedicine services was conducted. The COVID-19 Yorkshire Rehabilitation Scale was administered using an online survey 12 weeks after the COVID-19 infection. Prevalence of symptom severity and functional impairments were assessed to identify burdens and patterns. The correlation between symptom severity, functional impairments, patient characteristics, and overall health was evaluated. Results: The mean age was 46.8 years, with 46 (34.1%) males. Symptoms, functional impairments, and overall health worsened significantly when compared to the status prior to the infection. More than 50% reported the following sequelae 12 weeks after the acute infection: breathlessness, laryngeal or airway complications, fatigue, weakness, sleep, cognition, and anxiety. The presence of a single symptom or functional impairment significantly correlated with at least seven other problems positively, except for pain. Severity tended to be higher among vulnerable groups, including those who were chronic disease patients, older, less well educated, female, or had incomplete COVID-19 vaccinations. Conclusions: Long COVID is a significant healthcare burden among telemedicine users in Hong Kong, with complex needs for symptom and functional impairment management. Designing relevant health and rehabilitation services tailored to the needs of these patients is warranted.

## 1. Introduction

Post-COVID-19 Syndrome (Long COVID) is a heterogeneous, multi-system, relapsing, and remitting illness that can affect COVID-19 patients regardless of the severity of the initial COVID-19 [1,2]. The World Health Organization (WHO) proposes a clinical case definition in which Long COVID persists for three months from the onset of probable or confirmed COVID-19 [2]. Common symptoms include fatigue, shortness of breath, cognitive impairment, and other symptoms that often interfere with daily functioning [2]. These symptoms can persist, fluctuate, or relapse following the initial COVID-19 episode, or can newly appear after the resolution of acute COVID-19 [2]. For affected patients, these prolonged symptoms are also associated with functional impairments and possible frequent healthcare utilization [1,2,3].

Globally, the reported range of Long COVID incidence varies from 10% to 35 [4,5,6]. Despite the emergence and persistence of symptoms after acute COVID-19 infection being suggested to be a major burden on the healthcare system [7], there is very limited information on Long COVID prevalence, patterns, and clinical severity in Asian populations. To address this knowledge gap, this study aimed to assess the presence and features of Long COVID among a sample of COVID-19 patients during the fifth major wave in Hong Kong (starting from 31 December 2021), with the use of the Chinese version of the COVID-19 Yorkshire Rehabilitation Scale (C19-YRS). 

C19-YRS is a reliable and valid patient-reported outcome measure for assessing the symptom burden and severity of Long COVID comprehensively [8,9]. Its development and iterative changes involved both Long COVID patients’ and clinicians’ inputs [9]. The items of C19-YRS have covered all components of the 2001 WHO International Classification of Functioning, Impairment, and Health Framework [9]. The scale has shown its content validity in reliably capturing the emergence and persistent symptoms in existing studies [10,11,12]. C19-YRS has been recommended by United Kingdom The National Institute for Health and Care Excellence (UK NICE ) guideline and the National Health Service (NHS) England Clinical Guidance as a health needs assessment tool for individuals who recovered from acute COVID-19 infection [13,14].

## 2. Methods

### 2.1. Setting and Participants 

This cross-sectional study was conducted within a traditional Chinese medicine (TCM) telemedicine service setting [15]. The target population of this study was individuals who experienced a COVID-19 infection in Hong Kong, while our accessible sampling frame was composed of 337 adult users of the TCM telemedicine service who were diagnosed with COVID-19. The diagnosis should be confirmed using a polymerase chain reaction-based nucleic acid test or a rapid antigen test during February–April 2022. To align with WHO’s definition of Long COVID [2], patients were prompted to complete an online self-report version of C19-YRS 12 weeks after the COVID-19 infection. To increase the response rate, reminders for completing the online C19-YRS survey were sent out regularly, and patients’ inquiries about the study and survey questions were followed up timely. The Survey and Behavioural Research Ethics Committee at The Chinese University of Hong Kong granted ethical approval for this survey (reference no. SBRE-21-0589). Participants were informed of the study details and gave consent prior to online data collection.

### 2.2. C19-YRS Assessment

We obtained authorization from the original C19-YRS developers to translate the instrument from English to Cantonese for studying Long COVID in Hong Kong. The Cantonese version of C19-YRS was used in this study and administered online using REDCap, a secure web application for building and managing online surveys and databases [16]. C19-YRS consists of two main sections [9]:

The first section contains information about the respondent’s age, gender, and usage of health services after a COVID-19 infection. 

The second section investigates the presence of symptoms and functional impairments of Long COVID as listed in the C19-YRS tool. 

The severity of newly emerging and pre-existing symptoms and functional impairments are rated by the respondent from 0 to 10, where 0 indicates the absence of the symptom or functional impairment and 10 indicates that it is severe and life disturbing. To provide a baseline for comparison, both pre- and post-COVID infection statuses were assessed in this cross-sectional study. Current and pre-COVID self-reported health status was asked on a scale from 0 to 10, where 0 means the worst health the respondent can imagine and 10 means the best health one can imagine. Moreover, the current and pre-COVID employment status of the respondent was assessed. 

Based on the responses to C19-YRS, the respondents were stratified into one of the three overall severity categories (“severe”: 6–10 scores, “moderate”: 3–5.9 scores, or “mild”: 0–2.9 scores) [1]. This stratification was conducted separately for symptoms and functional impairments, based on the overall severity of the 15 most prevalent symptoms (except urinary or fecal incontinence) and all of the five functional impairments, respectively. Radar plots were drawn to show the mean individual scores of each symptom or functional impairment among respondents in each of the three overall severity categories. 

### 2.3. Statistical Analysis

Data analysis was performed using SPSS 23.0 (SPSS, Chicago, IL, USA) and graphs were produced using GraphPad Prism 7. Continuous data were expressed as mean ± standard deviation (SD), and categorical data were given as frequencies and percentages. The Wilcoxon Signed Ranks Test was used to compare scores before and after the acute COVID-19 infection. The Mann–Whitney U test was used for comparison between two groups in subgroup analyses. The Spearman rank correlation was used to explore the correlation between symptom severity, functional impairments, and overall health. All statistical tests were two-sided, and *p* < 0.05 was considered statistically significant.

## 3. Results

### 3.1. Characteristics of Respondents

A total of 135 patients (46 males and 89 females) completed the survey, and the response rate was 40.06%. The average age of patients was 46.78 ± 14.0 years, with a mean BMI of 23.06 ± 4.03 kg/m^2^. The demographic characteristics and clinical characteristics of patients were described in detail in Table 1. Among them, 85 patients (62.96%) received undergraduate education or above, and 9 patients (6.67%) were smokers. Ninety-one (67.41%) of the patients were still employed or studying as they did before COVID-19 infection, and thirty-seven (27.41%) stayed in the same role as being retired, homemakers, or unemployed. Only three patients (2.22%) reduced their working hours and one patient (0.74%) stopped working after a COVID-19 infection. Almost all participants (97.78%) were not hospitalized due to COVID-19, 48 patients (35.56%) reported having one or more chronic conditions, and 41 patients (30.37%) received 3 doses of COVID-19 vaccines.

### 3.2. Symptom and Functional Assessment

A total of 130 (96.30%) respondents complained of at least one symptom or functional impairment related to Long COVID. As presented in Table 2, symptoms, functional impairments, and overall health worsened significantly when compared to the status prior to the infection. More than 50% reported the following sequelae 12 weeks after COVID-19 infection: breathlessness (80.00%), fatigue (76.30%), laryngeal or airway complications (68.15%), cognition (57.46%), weakness (54.96%), sleep (52.67%), and anxiety (50.38%). Among them, the pre–post severity scores of breathlessness (1.60 ± 1.79 vs. 0.90 ± 1.23), laryngeal or airway complications (3.21 ± 2.41 vs. 0.72 ± 1.31), fatigue (3.57 ± 2.79 vs. 1.70 ± 1.71), and cognition (4.80 ± 2.15 vs. 1.77 ± 1.34) increased significantly (all *p* < 0.001). In addition, the overall self-perceived health score decreased from 6.78 ± 2.22 before acute COVID-19 to 5.43 ± 2.33 (*p* < 0.001) post-COVID-19. Among patients with laryngeal/airway complications, cough was the most common symptom (91.01%). Chest pain and headache were the most frequently reported among patients with pain, both at 23.53%. Among patients with cognition impairment, short-term memory problems accounted for 68.83% of the cases, which was the most common complaint.

### 3.3. Subgroup Analysis

To further explore the factors associated with the severity of Long COVID, we performed subgroup analyses by age, gender, education, chronic disease, and COVID-19 vaccination status. As shown in Table 3, symptoms and functional impairments were significantly more severe compared to pre-COVID-19 status in the following subgroups: more severe swallowing, depression, personal care and social function problems among those who are older than 50 years; lower social functions among females; lower personal care capability among those with secondary education or below; higher pain and cognition impairment among chronic disease patients; and finally, poorer mobility among those who received 0–2 doses of COVID-19 vaccines. The two main types of vaccines available in Hong Kong are Comirnaty (BNT 162b2) from Fosun Pharma/BioNTech and CoronaVac from Sinovac. 

### 3.4. Symptom and Functional Distribution in Three Severity Categories

**Figure 1:**
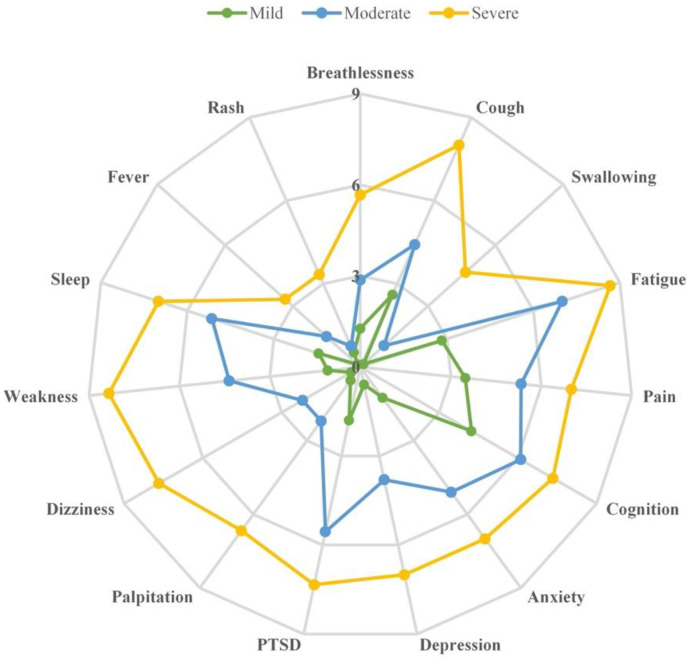
Radar plot of the mean severity of the 15 most prevalent Long COVID symptoms within each overall severity category, scored from 0 to 10. Notes: Based on the overall severity of the 15 symptoms, the three overall Long COVID severity categories were “severe” (with a mean symptom score of 6–10 scores), “moderate” (with a mean symptom score of 3–5.9 scores), and “mild” (with a mean symptom score of 0–2.9 scores).

Figure 1 showed that the numbers of individuals with mild, moderate, or severe overall symptom severity were 112, 20, and 3, respectively. Typically, the greater the overall severity the patients had, the higher the mean individual score for a specific symptom. In other words, patients in the severe overall severity category had higher mean individual scores for each of the 15 individual symptoms than that of those who were in the category of moderate or mild overall symptom severity. Figure 1 also indicated that while fatigue was the most serious symptom among patients in the category of severe overall severity (mean score of fatigue: 7.00) and moderate overall severity (mean score of fatigue: 8.67), cognition (mean score: 4.23) was the most disturbing symptom among patients with mild overall severity.

**Figure 2:**
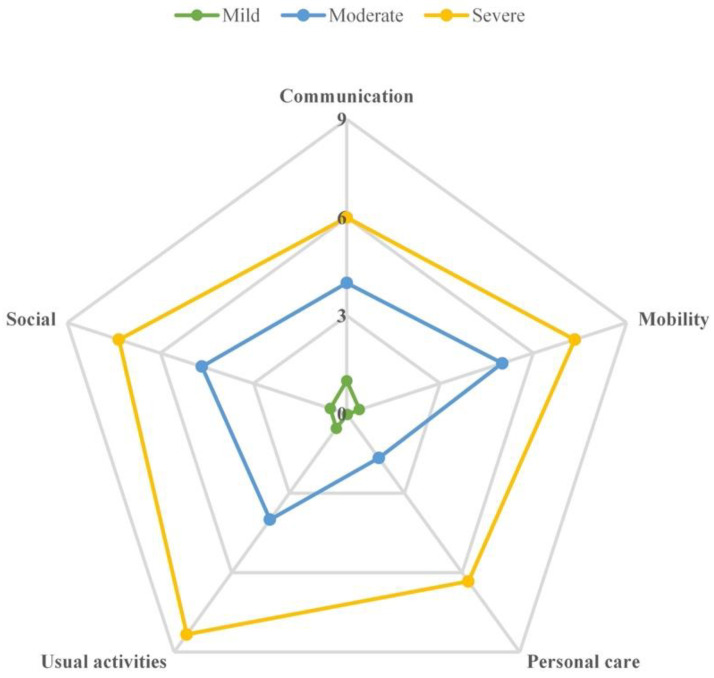
Radar plot of the mean severity of five Long COVID-related functional difficulties within each overall severity category, scored from 0 to 10. Notes: Based on the overall severity of the five functional difficulties, the three overall severity categories were “severe” (with a mean functional difficulty score of 6–10 scores), “moderate” (with a mean functional difficulty score of 3–5.9 scores), and “mild” (with a mean functional difficulty score of 0–2.9 scores).

Figure 2 revealed a similar distribution of functional impairment severity prevalence as Figure 1 did, with individuals with mild overall functional impairments being the majority (mild: 126, moderate: 3, severe: 3). In addition, the greater the overall severity patients had, the higher the mean individual score for a specific functional impairment. Among the five functional impairments assessed, the most affected domain was usual activities for individuals with severe and moderate overall severity, as reflected in the mean scores of 8.33 and 4.00, respectively.

### 3.5. Correlation Analysis between Symptom Severity, Functional Impairments, and Overall Health

**Figure 3:**
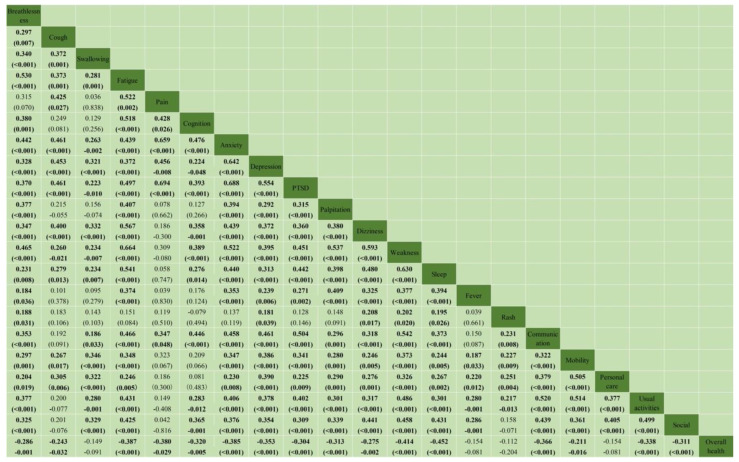
The correlation between symptom severity, functional disability, and overall health. Notes: All correlation was described as Spearman’s rank correlation coefficient (rs) and *p*-value. rs (*p*-values) with bold indicated statistically significant correlations; PTSD: Post-traumatic stress disorders.

As shown in Figure 3, the presence of a single symptom or functional impairment was significantly and positively correlated with at least seven other problems, except for pain. Among them, the combinations between two variables with correlation coefficients greater than 0.6 were: fatigue and weakness (rs = 0.664, *p* < 0.001), pain and anxiety (rs = 0.659, *p* < 0.001), pain and PTSD (rs = 0.694, *p* < 0.001), anxiety and depression (rs = 0.642, *p* < 0.001), anxiety and PTSD (rs = 0.688, *p* < 0.001), and weakness and sleep (rs = 0.630, *p* < 0.001). The overall health score was significantly and negatively associated with most symptoms and functional impairments (*p* < 0.05), except for swallowing, fever, rash, and personal care impairment (*p* > 0.05).

## 4. Discussion

We reported the prevalence, patterns, and clinical severity of Long COVID symptoms and functional impairments among COVID patients in Hong Kong. Our sample mainly consisted of non-hospitalized patients with a relatively mild initial COVID-19 infection, but almost all respondents suffered from varying degrees of Long COVID symptoms or functional impairments three months after the acute infection. This finding implied that the risk of developing Long COVID and its severity are irrelevant to hospitalization status during acute COVID, aligning with observations from previous studies [1,17,18,19]. Even though smoking and comorbidities were found to be risk factors for Long COVID [20], the prevalence of the sequelae remained high among our relatively healthy sample, of whom the majority were free from existing chronic diseases or smoking habits. 

The key symptoms of Long COVID that appeared in our study, including breathlessness, fatigue, laryngeal and airway complications, cognition, weakness, sleep problems, and anxiety, were akin to those reported in previous studies [21,22,23,24]. It is worth noting that the prevalence of these symptoms may increase substantially over time [23]. The pathophysiology of Long COVID is still enigmatic, and there is a wide range of putative mechanisms proposed, including long-term organ damage due to acute-phase infection, ongoing and sustained inflammatory response, immune dysregulation, autoimmunity, endothelial dysfunction, occult viral persistence, as well as coagulation activation [25,26]. Although these proposed mechanisms require further investigation, the likely multifaceted mechanisms in Long COVID pathogenesis could partly explain its persistent, heterogeneous, and fluctuating nature.

Complex pathogenesis may also explain the co-existence of multiple symptoms and functional impairments among Long COVID patients. In alignment with findings from a previous C19-YRS-based survey [1], we also found strong correlations between Long COVID symptoms, functional impairments, and self-perceived overall health status. Our study discovered that the presence of a single symptom or functional impairment was significantly and positively correlated with at least seven other problems. For moderate to severe cases, a current guideline suggests that the management of this complex, multifaceted clinical condition requires multidisciplinary approaches [27]. As recommended by the UK National Institute of Clinical Excellence, a multidisciplinary team with expertise in physical, psychological, and psychiatric services is needed to investigate and manage a wide range of Long COVID presentations [27]. It is likely that the healthcare model for Long COVID will need to be adapted in accordance with the health system context. Our sample is derived from TCM telemedicine service users, and they may choose to use TCM as one of the options for managing their Long COVID as well. In fact, the public health system of Hong Kong has provided free TCM out-patient rehabilitation services for post-COVID patients since April 2020 [28]. The effectiveness of TCM, including Chinese herbal medicine, acupuncture, and therapeutic massage, remained unclear until rigorous service evaluation or randomized trials were conducted. 

Long COVID can also cause new or worsening difficulties in completing activities of daily living, or in hindering a return to work. In our study, although more than one-fourth of our respondents reported new or worsening difficulties in completing activities of daily living, surprisingly, their employment status was only affected in 2.22% of cases. This is a relatively low figure in contrast with results from a U.S. study, in which 40% of patients could not return to work because of ongoing health issues or job loss [29]. Indeed, our results showed that the majority of Long COVID is of a mild nature, and current guidelines [27] and care models [30] recommend online self-management support as one of the first-line interventions for managing Long COVID, prior to health service utilization. This recommendation has been implemented in the NHS England Long COVID stepped-care pathway, in which all patients with typical Long COVID symptoms are first signposted to an online self-management program [31]. Hong Kong has recently launched similar initiatives as well [32].

Nevertheless, our study found that some patient subgroups might have more severe Long COVID symptoms and functional impairments. Similar to other studies, aging and pre-existing chronic conditions were also found to be associated with the development of Long COVID [20]. Female participants in our study were more severely affected with respect to social roles than male participants. A previous study found that females had more Long COVID symptoms, such as new-onset pain, airway complaints, concentration, and short-term memory impairment, than males [3]. Other studies indicated that the female gender was linked to a higher risk of developing Long COVID [20,33]. In addition, patients with lower education levels also appeared to be more susceptible to a higher Long COVID burden in our study. Policy to ensure timely and fair access to appropriate health service across all patients of different socioeconomic statuses and genders is important for achieving health equity [34]. 

Our subgroup analysis implies that clinicians should pay particular attention to these susceptible and vulnerable groups early on at the time of making a diagnosis of COVID-19 infection, such that a follow-up Long COVID assessment can be conducted to facilitate appropriate triage. The C19-YRS was found to be easy and simple to use even for people without previous training [3], and our experience echoes such an observation. It is suggested that C19-YRS can be used as a Long COVID screening tool during follow-up in Hong Kong as well. In Hong Kong, from 24 February 2022 onward, all persons entering or remaining on the specified premises, such as restaurants, fitness centers, and places of public entertainment, are required to receive at least three doses of COVID-19 vaccines [35]. This was the probable reason why respondents with incomplete vaccination felt more affected in terms of mobility compared with those fully vaccinated.

This study has several limitations. First, the cross-sectional design prevented us from reaching a causal conclusion. A cohort study is required to determine whether the observed associations between the severity of Long COVID symptoms and functional impairments and demographic or health factors are causal. A cohort study with a larger sample size should be conducted, which will allow the use of multivariate analyses for examining such potential causal relationships. For example, there is a need to further assess the potential preventative effect of the COVID vaccine administered prior to acute SARS-CoV-2 infection on the subsequent development of Long COVID symptoms [36]. Second, respondents were asked to recall the severity of their symptoms and functional impairments prior to their COVID-19 infection, hence, there is a possibility of recall bias in estimating the pre-post differences. Third, while the use of the Chinese C19-YRS enabled us to collect comprehensive data on Long COVID presentations, a future survey should use the updated, modified version of the C19-YRS (C19-YRSm) [37]. A formal assessment of the psychometric characteristics of the Chinese version of C19-YRSm should be conducted. Fourth, our study has a mediocre response rate (40.06%) [38]. Nonrespondents’ reasons for not participating and their characteristics were not compared to those of respondents due to a lack of data, raising the possibility of non-response bias. Fifth, as this study sampled users of Chinese medicine telemedicine services, this may limit the representativeness of our sample. That said, we invited all service users to participate without selection. This protected our survey from selection bias and allowed estimation of the Long COVID disease burden among typical primary care service users in Hong Kong. Finally, as the pandemic continues, telemedicine remains to be a key strategy to facilitate timely access to healthcare services worldwide [39]. With a focus on Long COVID, we did not investigate other important ethical and legal issues related to telemedicine practice in this survey. Future studies on the key quality issues in enhancing patient safety, privacy protection, informed consent process, and patient satisfaction are warranted [40].

## 5. Conclusions

Our study found that Long COVID symptoms and functional impairments are highly prevalent among non-hospitalized COVID patients in Hong Kong. The most prevalent sequelae 12 weeks after the acute COVID infection included breathlessness, laryngeal or airway complications, fatigue, weakness, sleep disturbance, cognition impairments, and anxiety. The presence of a single symptom or functional impairment is positively and significantly correlated with at least seven other problems, except for pain. The severity of symptoms and functional impairments tends to be higher among vulnerable groups, including those who are chronic disease patients, older, females, or less well educated. Our study contributes to a better understanding of the prevalence, patterns, and clinical severity of various Long COVID symptoms and functional impairments, which may aid health authorities in better designing, providing, and resourcing relevant health and rehabilitation services tailored to the needs of Long COVID patients, particularly vulnerable individuals. 

## Figures and Tables

**Table 1:** Baseline characteristics of respondents.

Demographic Characteristics	All Respondents (n = 135)
Age (yr)	46.78 ± 14.05
Gender (male)	46 (34.07%)
BMI (kg/m^2^)	23.06 ± 4.03
Degree of education	
High school or below	37 (27.41%)
Undergraduate or above	85 (62.96%)
Not reported	13 (9.63%)
Smoking ^a^	9 (6.67%)
Employment status	
Still employed/student	91 (67.41%)
Still retired/homemaker/unemployed	37 (27.41%)
Reduced hours	3 (2.22%)
Stopped work	1 (0.74%)
Not reported	3 (2.22%)
Clinical characteristics	
Admission to hospital	3 (2.22%)
Other medical	48 (35.56%)
Number of chronic diseases	
0	73 (54.07%)
1	33 (24.44%)
2	13 (9.63%)
3	3 (2.22%)
Not reported	13 (9.63%)
COVID-19 vaccine doses	
0	3 (2.22%)
1	12 (8.89%)
2	66 (48.89%)
3	41 (30.37%)
Not reported	13 (9.63%)
Received influenza vaccination in the past 3 months	10 (7.41%)

Note: All data were expressed as n (percentage) or means ± standard deviation (SD); ^a^ Smoking was defined as smoking more than 100 cigarettes during the lifetime.

**Table 2:** Prevalence and scores of symptoms, functional difficulties, and overall health as measured using C19-YRS.

Symptoms/Functional Impairments	N	Prevalence ^a^		Score ^b^			
		Current	Pre-COVID	Current	Pre-COVID	*Z*	*p*
**Breathlessness**	135	108 (80.00%)	93 (68.89%)	1.60 ± 1.79	0.90 ± 1.23	−6.907	**<0.001**
At rest	135	42 (31.11%)	21 (15.56%)	1.05 ± 1.95	0.39 ± 1.15	−5.203	**<0.001**
On dressing	130	24 (18.46%)	11 (8.46%)	0.61 ± 1.61	0.28 ± 1.07	−3.757	**<0.001**
On walking	128	103 (80.47%)	93 (72.66%)	3.13 ± 2.47	2.09 ± 1.94	−6.562	**<0.001**
**Laryngeal/airway complications**	135	92 (68.15%)	-	3.21 ± 2.41	0.72 ± 1.31	−7.514	**<0.001**
Cough	135	86 (63.70%)	-	-	-	-	-
Voice	135	23 (17.04%)	-	-	-	-	-
Breathing murmur	135	10 (7.41%)	-	-	-	-	-
The worst among them							
cough	89	81 (91.01%)	-	3.19 ± 2.43	0.78 ± 1.35	−7.155	**<0.001**
voice	89	5 (5.62%)	-	4.20 ± 2.59	0.20 ± 0.45	−1.841	0.066
breathing murmur	89	3 (3.37%)	-	2.33 ± 1.16	0.00 ± 0.00	−1.633	0.102
**Swallowing**	135	19 (14.07%)	9 (6.67%)	0.38 ± 1.18	0.19 ± 0.83	−3.482	**<0.001**
Nutrition	135	15 (11.11%)	-	-	-	-	-
**Fatigue**	135	103 (76.30%)	89 (65.93%)	3.57 ± 2.79	1.70 ± 1.71	−8.254	**<0.001**
**Incontinence**	134	6 (4.48%)	-	5.33 ± 3.39	2.33 ± 1.51	−2.041	**0.041**
Fecal	134	1 (0.75%)	-	-	-	-	-
Urinary	134	6 (4.48%)	-	-	-	-	-
The worst among them							
fecal	6	0 (0.0%)	-	-	-		
urinary	6	6 (100.0%)	-	5.33 ± 3.39	2.33 ± 1.51	−2.041	**0.041**
**Pain**	134	34 (25.37%)	-	4.18 ± 1.99	0.82 ± 1.40	−4.961	**<0.001**
Chest pain	134	12 (8.96%)	-	-	-	-	-
Joint pain	134	11 (8.21%)	-	-	-	-	-
Muscle ache	134	17 (12.69%)	-	-	-	-	-
Headache	134	17 (12.69%)	-	-	-	-	-
Abdominal pain	134	8 (5.97%)	-	-	-	-	-
Other pain	134	8 (5.97%)	-	-	-	-	-
The worst in the past week							
chest pain	34	8 (23.53%)	-	4.13 ± 1.46	0.50 ± 0.76	−2.539	**0.011**
joint pain	34	4 (11.76%)	-	6.00 ± 2.58	2.25 ± 2.22	−1.890	0.059
muscle ache	34	7 (20.59%)	-	4.71 ± 1.70	0.86 ± 1.57	−2.226	**0.026**
headache	34	8 (23.53%)	-	3.63 ± 2.13	1.00 ± 1.60	−2.539	**0.011**
abdominal pain	34	3 (8.82%)	-	4.00 ± 1.00	0.00 ± 0.00	−1.604	0.109
other pain	34	4 (11.76%)	-	2.75 ± 2.50	0.25 ± 0.50	−1.604	0.109
**Cognition**	134	77 (57.46%)	-	4.80 ± 2.15	1.77 ± 1.34	−7.355	**<0.001**
Concentrating	134	53 (39.55%)	-	-	-	-	-
Short-term memory	134	72 (53.73%)	-	-	-	-	-
Event planning	134	38 (28.36%)	-	-	-	-	-
The worst among them							
concentrating	77	20 (25.97%)	-	4.15 ± 1.63	1.55 ± 1.15	−3.759	**<0.001**
short-term memory	77	53 (68.83%)	-	4.96 ± 2.25	1.75 ± 1.31	−6.177	**<0.001**
event planning	77	4 (5.19%)	-	6.00 ± 2.94	1.75 ± 1.26	−1.633	0.102
**Anxiety**	133	67 (50.38%)	55 (41.35%)	1.96 ± 2.46	1.02 ± 1.58	−6.075	**<0.001**
**Depression**	133	47 (35.34%)	41 (30.83%)	1.22 ± 2.13	0.70 ± 1.43	−4.669	**<0.001**
Thoughts about harming yourself	133	2 (1.50%)	-	-	-	-	-
**Post-traumatic stress disorder**							
Unwanted memories	134	7 (5.22%)	-	-	-	-	-
Unpleasant dreams	134	8 (5.97%)	-	-	-	-	-
Avoid thoughts	134	18 (13.43%)	-	-	-	-	-
Pressure	133	84 (63.16%)	78 (58.65%)	2.49 ± 2.52	1.74 ± 1.92	−5.968	**<0.001**
**Communication**	132	45 (34.09%)	36 (27.27%)	1.19 ± 1.97	0.56 ± 1.23	−5.050	**<0.001**
**Mobility**	132	27 (20.45%)	14 (10.61%)	0.67 ± 1.61	0.32 ± 1.09	−4.250	**<0.001**
**Personal care**	132	7 (5.30%)	4 (3.03%)	0.20 ± 1.05	0.14 ± 0.86	−2.271	**0.023**
**Usual activities**	132	34 (25.76%)	16 (12.12%)	0.80 ± 1.76	0.28 ± 1.07	−4.743	**<0.001**
**Social**	132	30 (22.73%)	15 (11.36%)	0.78 ± 1.82	0.26 ± 0.98	−4.408	**<0.001**
**Palpitation**	132	40 (30.30%)	-	0.94 ± 1.70	-	-	-
**Dizziness**	131	42 (32.06%)	-	0.80 ± 1.59	-	-	-
**Weakness**	131	72 (54.96%)	-	1.75 ± 2.18	-	-	-
**Sleep**	131	69 (52.67%)	-	2.14 ± 2.77	-	-	-
**Fever**	131	19 (14.50%)	-	0.40 ± 1.24	-	-	-
**Rash**	131	27 (20.61%)	-	0.62 ± 1.47	-	-	-
**Overall health**	130	-	-	5.43 ± 2.33	6.78 ± 2.22	−8.008	**<0.001**

Note: ^a^ The data were expressed as n (percentage); ^b^ The data were expressed as means ± standard deviation (SD); *p* values with bold were considered statistically significant differences; - Not applicable.

**Table 3:** Subgroup analysis for changed scores of symptoms, functional difficulties, and overall health measured using C19-YRS.

	Age		Gender		Education		Chronic Disease		COVID-19 Vaccine	
	*Z*	*p*	*Z*	*p*	*Z*	*p*	*Z*	*p*	*Z*	*p*
Breathlessness	−0.824	0.410	−1.061	0.288	−0.864	0.387	−0.404	0.686	−1.278	0.201
Cough	−0.703	0.482	−0.546	0.585	−1.301	0.193	−0.464	0.643	−0.408	0.684
Swallowing	−2.788	**0.005** ^b^	−0.758	0.449	−0.372	0.710	−0.758	0.448	−0.740	0.459
Fatigue	−0.858	0.391	−1.107	0.268	−1.274	0.203	−0.306	0.760	−0.203	0.839
Pain	−0.562	0.574	−1.173	0.241	−0.075	0.940	−2.685	**0.007** ^a^	−1.450	0.147
Cognition	−0.515	0.606	−0.360	0.719	−0.125	0.901	−1.992	**0.046** ^a^	−0.656	0.512
Anxiety	−0.674	0.500	−1.258	0.208	−0.405	0.686	−0.650	0.516	−1.907	0.056
Depression	−2.195	**0.028** ^b^	−1.332	0.183	−0.153	0.879	−0.397	0.691	−1.117	0.264
PTSD	−0.847	0.397	−0.278	0.781	−0.597	0.551	−0.085	0.932	−1.592	0.111
Communication	−0.640	0.522	−0.717	0.474	−0.970	0.332	−0.104	0.917	−0.223	0.824
Mobility	−1.544	0.123	−0.519	0.604	−0.986	0.324	−1.435	0.151	−1.994	**0.046** ^a^
Personal care	−3.097	**0.002** ^b^	−0.027	0.979	−3.069	**0.002** ^a^	−1.422	0.155	−0.387	0.699
Usual activities	−1.081	0.280	−1.657	0.098	−0.703	0.482	−0.696	0.486	−1.293	0.196
Social	−2.276	**0.023** ^b^	−2.098	**0.036** ^b^	−0.804	0.422	−1.784	0.074	−0.735	0.463
Overall health	−0.527	0.598	−0.167	0.867	−0.382	0.702	−0.570	0.569	−1.054	0.292

Note: *p* values in bold were considered as indicating statistically significant differences; Subgroup 1: Age = under the age of 50, Gender = Male, Education = High school or below, Chronic disease = with chronic disease, COVID-19 vaccine = 0–2 doses; Subgroup 2: Age = over the age of 50, Gender = Female, Education = Undergraduate or above, Chronic disease = without chronic disease, COVID-19 vaccine = 3 doses; ^a^ Subgroup 1 scored higher than subgroup 2, indicating relatively higher severity among subgroup 1; ^b^ Subgroup 2 scored higher than subgroup 1, indicating relatively higher severity among subgroup 2; PTSD: Post-traumatic Stress Disorder.

## Data Availability

The data that has been used is confidential and cannot be shared.

## Abbreviations

C19-YRS: COVID-19 Yorkshire Rehabilitation Scale; Long COVID: Post-COVID-19 Syndrome; NHS: National Health Service; UK NICE: United Kingdom The National Institute for Health and Care Excellence; WHO: World Health Organization
